# Incidence and risk factors for recurrent IgA nephropathy after kidney transplantation: A multicenter cohort study from Denmark

**DOI:** 10.1371/journal.pone.0353887

**Published:** 2026-07-20

**Authors:** Mette Thrane Øhrstrøm, Michael Dyrehauge Schultz, Claus Bistrup, Henrik Birn

**Affiliations:** 1 Department of Renal Medicine, Aarhus University Hospital, Aarhus, Denmark; 2 Department of Nephrology, Odense University Hospital, Odense, Denmark; 3 Department of Clinical Research, University of Southern Denmark, Odense, Denmark; 4 Department of Clinical Medicine, Aarhus University, Aarhus, Denmark; Sultan Qaboos University College of Medicine and Health Science, OMAN

## Abstract

**Background:**

Recurrent immunoglobulin A nephropathy after kidney transplantation remains a major clinical challenge, but risk factors are not fully defined. This study aimed to evaluate the incidence of biopsy-proven recurrent IgAN and identify risk factors for recurrence in a contemporary Scandinavian cohort.

**Methods:**

We conducted a multicenter cohort study including adult patients with biopsy-proven immunoglobulin A nephropathy who received a first kidney-only transplantation between 1990 and 2020 across two Danish transplantation centers. Recurrence incidence and associations with clinical and donor-related characteristics were assessed during follow-up.

**Results:**

A total of 118 recipients were followed for a median of 5.5 years. Biopsy-confirmed recurrence occurred in 14% of patients, corresponding to a cumulative incidence of 16% at 10 years post-transplantation. Younger age at immunoglobulin A nephropathy diagnosis, faster progression to kidney failure, and receipt of a kidney from a living or genetically related donor were independently associated with an increased risk of recurrence. Maintenance post-transplant steroid use was not associated with recurrence. Recurrent immunoglobulin A nephropathy was associated with an increased risk of graft failure compared with non-recurrent disease (unadjusted HR 3.8; adjusted HR 6.7).

**Conclusions:**

Recurrence of IgAN after kidney transplantation remains a clinically significant challenge, associated with younger age, rapid progression to KF, and transplantation from living or genetically related donors. Maintenance post-transplant steroid use was not associated with recurrence risk in this cohort. Larger and more diverse cohorts are warranted to clarify donor-related risk, strengthen the evidence on immunosuppressive protocols and guide strategies to improve graft outcomes.

## Introduction

Immunoglobulin A nephropathy (IgAN) is the most common glomerulopathy worldwide and progresses to kidney failure (KF) in 30–50% of affected individuals [1–3].

Most patients are diagnosed before age 40, making many eligible for kidney transplantation during their lifetime [3,4]. IgAN accounts for 7–14% of all kidney transplants [5,6].

IgAN recurs in 9–52% of kidney allografts, partly depending on biopsy strategies [7]. Although not all recurrences lead to graft loss, a large retrospective registry study including 9,690 patients from the OPTN/UNOS database reported recurrent IgAN as the third most common cause of graft failure in patients with IgAN as the primary disease [8]. No effective treatment exists for recurrent IgAN, which makes the identification of risk factors crucial for prevention and improved outcomes [9].

Several factors have been associated with increased risk of IgAN recurrence, including donor type, young age, higher HLA-mismatch, and immunosuppressive regimens [10–12]. The potential role of donor type, particularly living and genetically related donors remains uncertain, as some studies have reported an increased risk of recurrence in these subgroups, while others have found no clear association [11,13–15]. The impact of maintenance post-transplant steroid use is also debated: some studies suggest a protective effect, whereas others report no association with recurrence risk [8,12,16–18]. These discrepancies may reflect differences in study populations and immunosuppressive protocols beyond steroid use. Geographical variation may also contribute, as IgAN is more prevalent in East Asia than in Europe and North America, which may influence recurrence patterns [19]. Also, and importantly, the rationale for choosing a post-transplant steroid-free immunosuppressive protocol is often unclear in previous studies, potentially leading to indication bias.

Further studies in diverse cohorts are needed to identify risk factors for IgAN recurrence after transplantation. Contemporary Scandinavian data remain limited, as earlier studies were small and conducted in earlier eras of immunosuppression [20,21].

In this Danish multicenter cohort study, we report the incidence of biopsy-proven recurrent IgAN after kidney transplantation, identify potential risk factors associated with recurrence, including the role of maintenance post-transplant steroids, and examine the association between recurrence and graft survival.

## Materials and methods

### Study design

An observational, cohort study, including all adult patients (≥ 18 years) with biopsy-proven IgAN and first kidney-only transplantation from 1990 to 2020 at Aarhus University Hospital or Odense University Hospital. The standard immunosuppression protocol at Aarhus University Hospital included maintenance prednisolone 5 mg/day from month 6; Odense used a steroid-free protocol until 2017. At both centers tacrolimus and mycophenolate were used as standard immunosuppressive therapy, and the choice of induction therapy was consistent, with either anti-thymocyte globulin (Thymogobuline) or anti-CD25 antibody (Basilixumab) being primarily used. The study was approved by the Regional Authority of the Region of South Denmark on behalf of the involved Danish regions (approval number: 21/21623). The requirement for individual informed consent, written or verbal, was waived in accordance with the Danish Health Care Act (§ 46, subsection 2).

### Study population

**Fig 1:**
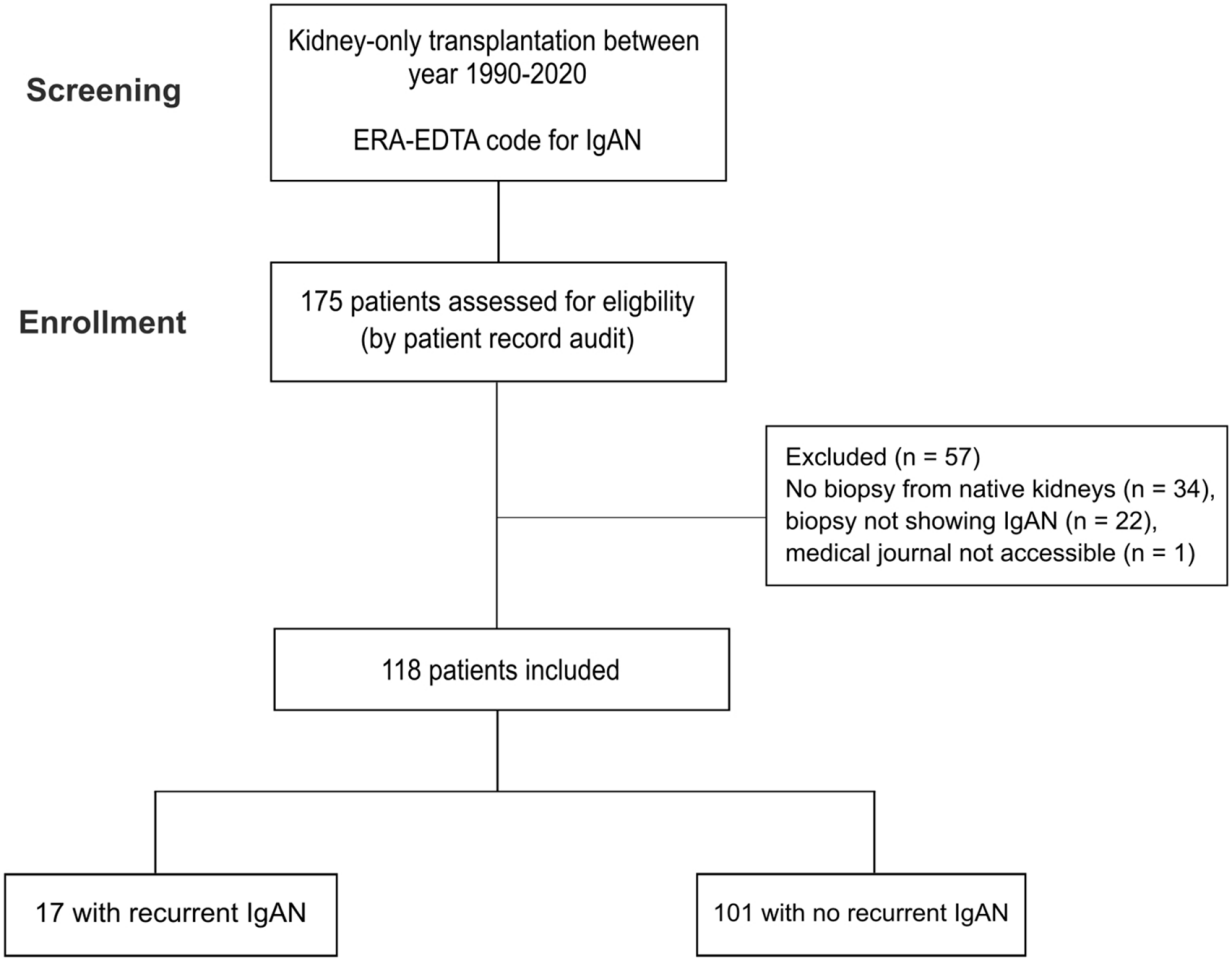
The study population enrollment. Flow diagram showing the inclusion of kidney transplant recipients with IgA nephropathy in the present study. ERA-EDTA, European Renal Association – European Dialysis and Transplant Association.

The study population enrollment is shown in Fig 1.

The patients were identified from the National Danish Renal Registry (Dansk Nefrologisk Selskabs Landsregister (DNSL), The Danish Clinical Quality Program, www.rkkp.dk) which includes all Danish kidney transplants since 1990. We initially identified first time renal transplant patients from 1990 to 2020 with one of the following ERA-EDTA (European Renal Association – European Dialysis and Transplant Association) codes for primary renal disease: IgA nephropathy, mesangial proliferative glomerulonephritis, focal and segmental proliferative glomerulonephritis, glomerulonephritis – secondary to systemic disease and Henoch-Schönlein purpura. The diagnosis of IgAN was verified by examination of all pathology reports and only kidney recipients with biopsy-proven IgAN in native kidneys were included. The study population was followed until graft failure, death, loss to follow-up or June 1, 2022.

### Definitions

Biopsy-proven IgAN was defined as predominant mesangial IgA deposition on immunofluorescence and/or as a mesangioproliferative glomerulonephritis with immune complex deposits identified by electron microscopy. The evaluation was performed by two authors and later by consensus among all authors before inclusion in the study. Recurrent IgAN was defined by the same criteria when identified in a kidney graft biopsy at any time. The date of the graft biopsy defined the time of recurrence. All kidney biopsies (native or graft) were clinically indicated; no protocol biopsies were performed. The Oxford MEST classification was not systematically reported in the pathology records throughout the study period and was therefore not available for consistent analysis in this cohort.

Kidney failure (KF) refers to the stage of native kidney disease requiring kidney replacement therapy (dialysis or transplantation). Graft failure was defined as time of re-transplantation, start of dialysis or death with a functioning graft.

Donor type was examined in two ways: by donor status (living vs. deceased) and by genetic relation (genetically related vs. non-related). The non-related group included both living unrelated and deceased donors.

### Data collection and outcome

Baseline data and outcome were extracted from DNSL and electronic patient records. Variables included demographics, smoking, comorbidity, dialysis history, age at IgAN diagnosis, death, HLA mismatch (A, B, DR), ABO compatibility, donor type, cold ischemia time, induction therapy, maintenance steroid use, biopsy-proven rejection and recurrence, and time/cause of graft failure. Steroid use was assessed annually after transplantation and quantitated as total time of steroid treatment in years.

The data were accessed for research purposes from June 2022 onwards. During data collection, only the authors involved in data extraction had access to identifiable patient information within secure hospital system, in accordance with the ethics approval. Other authors did not have access to identifiable data.

The primary outcome of the study was biopsy-proven recurrent IgAN, while secondary outcomes were graft failure, death and rejections.

### Statistics

Baseline categorical data are presented as frequencies and percentages. Continuous variables are presented as medians with interquartile range (IQR) or means with standard deviations (SD) as appropriate.

Risk factors for recurrence were analyzed using univariable and multivariable cox regression and expressed as hazard ratios (HR) with 95% confidence interval (95% CI). The multivariable model adjusted for sex and age at KF.

Cumulative risks for recurrence by donor type are presented as Aalen-Johansen plots.

The risk of graft failure was analyzed using cox regression, with recurrent IgAN treated as a time-varying covariate, and results reported as HR with 95% CI. 5- and 10-year death censored graft survival were also calculated.

The statistical analyses were performed using the statistical software Stata version 18.0 for Windows (StataCorp LLC), while Aalen-Johansen plots were generated using RStudio (v. 2023.12.1 + 402).

## Results

### Study population

A total of 118 recipients with IgAN as primary cause of KF underwent a first-time kidney transplantation during the study-period at the two transplantation centers in Denmark. 17/118 (14%) recipients were diagnosed with biopsy-proven recurrent IgAN, and 101/118 (86%) recipients were not. The median follow-up time was 5.52 years (IQR 2.83−9.33) equivalent to 798 patient-years.

### Baseline characteristics

Baseline demographics and clinical characteristics are shown in Table 1.

**Table 1:** Baseline demographics and clinical characteristics of kidney transplant recipients with IgAN.

N = 118	All patients (N = 118)	Recurrent IgAN (N = 17)	Non-recurrent IgAN (N = 101)	p-value^b^
**Baseline characteristics**				
Male sex (%)	81 (69%)	11 (65%)	70 (69%)	0.705
Follow-up time, years (median (IQR))	5.52 (2.83-9.33)	6.04 (3.20-10.37)	5.37 (2.80-9.27)	0.4664
Age at time of Kidney failure, years (mean ±SD)	40.90 ± 13.84	30.65 ± 8.75	42.62 ± 13.83	0.0008
Time from diagnosis to Kidney failure, years (median (IQR))	3.53 (1.64−6.59)	1.88 (0.94−2.45)	3.66 (1.85−7.63)	0.0135
**Pretransplant comorbidity**				
Hypertension (%)	111/112 (99%)	17/17 (100%)	94/95 (99%)	0.535
Diabetes (%)	4/115 (3%)	1/17 (6%)	3/98 (3%)	0.648
Heart failure	3/114 (3%)	1/17 (6%)	2/97(2%)	0.462
Ischemic heart disease	6/115 /5%)	0/17 (0%)	6/98 (6%)	0.440
**Primary IgA-nephropathy, baseline characteristics**				
Age at time of diagnosis, years (mean ±SD)	35.80 ± 13.59	28.65 ± 9.01	37.00 ± 13.89	0.0184
Histological findings				
Crescents	33/118 (28%)	6/17 (35%)	27/101 (26%)	0.467
Mesangial proliferation	97/118 (82%)	14/17 (82%)	83/101 (82%)	0.986
Deposition of IgA on immunflourescence	109/118 (92%)	16/17 (94%)	93/101 (92%)	0.770
Immunodeposits on electron microscopy	74/118 (63%)	12/17 (71%)	62/101 (61%)	0.468
**Transplant characteristics**				
Living donor	58/118 (49%)	14/17 (82%)	44/101 (44%)	0.003
Living genetically related donor	47/118 (40%)	12/17 (71%)	35/101 (35%)	0.005
Living non-genetically related donor	11/118 (9%)	2/17 (12%)	9/101 (9%)	0.608
Pre-emptive transplantation, yes	94/118 (80%)	14/17 (82%)	80/101 (79%)	0.766
AB0-compability	104/118 (88%)	14/17 (82%)	90/101 (89%)	0.425
Donor specific antibodies positivity	5/118 (4%)	1/17 (6%)	4/101 (4%)	0.936
HLA zero mismatch	9/101 (8%)	1/16 (6%)	8/94 (9%)	0.760
Median number of HLA mismatch (min;max)	3 (0;6)	2.5 (0;5)	3 (0;6)	0.490
Cold ischemia time, hours (median (IQR))	8.22 (3.92−14.00)	3.82 (3.22−5.00)	9.12 (4.10−14.48)	0.0280
Induction therapy				0.660
Basiliximab	80/111 (72%)	12/16 (75%)	68/95 (72%)	
Thymoglobulin	17/111 (15%)	1/16 (6%)	16/95 (17%)	
Rituximab	5/111 (5%)	1/16 (6%)	4/95 (4%)	
Other^a^	9/111 (8%)	2/16 (13%)	7/95 (7%)	
Steroid, part of maintenance immunosuppression				
Yes	101/118 (85%)	16/17 (94%)	85/101 (84%)	0.279
Treatment years (median (IQR))	4.0 (1.0-8.0)	6.0 (2.0−12.0)	4.0 (1.0−8.0)	0.1107
**Post transplantation**				
Delayed graft function	9/118 (8%)	0/17 (0%)	9/101 (9%)	0.200
Acute rejection, humoral or cellular	24/118 (20%)	4/17 (24%)	20/101 (20%)	0.724
Time to biopsy-proven recurrent IgAN, years (median (IQR))		2.92 (0.89-5.60)		
Graft-failure	28/118 (24%)	10/17 (59%)	18/101 (18%)	0.000
Time to graft-failure, years (median (IQR))	5.06 (1.79−8.98)	4.13 (1.86−6.04)	5.22 (1.55−10.00)	0.9998
Death, yes / no	10/118 (9%)	1/17 (6%)	9/101 (9%)	0.678

Data are presented for the total cohort (N = 118) and stratified by recurrence status (recurrent vs. non-recurrent IgAN). Values are presented as n (%), mean ±SD, or median (interquartile range).

^a^Other induction therapy includes thymoglobulin plus rituximab (n = 4) and basiliximab plus rituximab (n = 5).

^b^P-values were calculated using the Pearson chi-square test for categorical variables and the Student’s t-test for continuous variables.

In the total cohort, 69% were male, and the mean age at the time of diagnosis was 35.8 years ± 13.6.

No major difference was found between patients with and without recurrent IgAN in terms of sex, comorbidities or histological findings on native kidney biopsy. However, the patients with recurrent IgAN were younger at time of IgAN diagnosis (recurrent IgAN: 28.7 years ± 9.0 vs non-recurrent IgAN 37.0 years ± 13.9, p = 0.0184), younger at KF (recurrent IgAN: 30.7 years ± 8.75 vs non-recurrent IgAN 42.6 years ± 13.8, p = 0.0008), and progressed faster from primary diagnosis to KF (recurrent IgAN: 1.9 years (IQR 0.9–2.5) vs non-recurrent IgAN 3.7 years (IQR 1.9–7.6), p = 0.0135).

### Transplant and immunosuppression characteristics

Of the 118 recipients, 58 (49%) received a kidney from a living donor. This included 47 (40%) with a living genetically related donor and 11 (9%) with a living non-genetically related donor. Living donor transplantation was more common in the recurrence group, where 14/17 (82%) received a kidney from a living donor, compared to 44/101 (44%) in the non-recurrence group. Living genetically related donors accounted for 12/17 (71%) of the recurrence group, compared to 35/101 (35%) in the non-recurrence group.

Most transplants were AB0-compatible. Pre-emptive transplantation was performed in 94/118 recipients (80%), and donor-specific antibodies at the time of transplantation were present in 5/120 recipients (4%). The median cold ischemia time was 8.2 hours (IQR 3.9–14.0) for all patients, 3.8 hours (IQR 3.2–5.0) in the recurrence group, and 9.1 hours (IQR 4.1–14.5) in the non-recurrence group.

A total of 101/118 recipients (85%) were on steroid-containing immunosuppression protocols with 16/17 (94%) in the recurrence group and 85/101 (84%) in the non-recurrence group. There was no difference in the use of induction therapy between groups.

### Incidence

The median time from transplantation to the biopsy defining recurrence of IgAN was 2.9 years (IQR 0.9−5.6).

**Fig 2:**
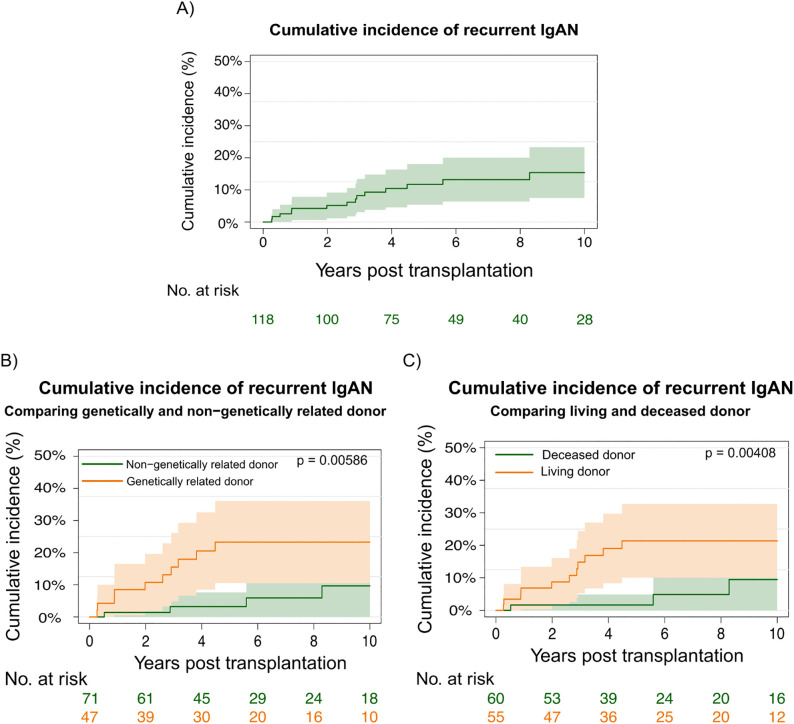
Time to event analysis from time of transplantation to recurrent IgAN. (A) Cumulative risk for recurrent IgAN for the whole study population 10 years post transplantation. (B + C) Cumulative risk for recurrent IgAN comparing donor type, B) genetically related donor vs. non-genetically related and (C) living vs. deceased donor, respectively.

The cumulative incidence of biopsy-proven recurrent IgAN at 1-, 5-, and 10-years post-transplantation was 4% (95% CI 1.8–9.8), 12% (95% CI 6.8–19.9), and 16% (95% CI 9.2–25.9), respectively (Fig 2A).

### Risk factors for recurrence

In unadjusted analyses, younger age at KF (HR 0,93 per year increase; 95% CI 0.89−0.98), shorter time from diagnosis to KF (HR 0.70 per year; 95% CI 0.54−0.91), donor type, and shorter cold ischemia time (HR 0.85 per hour increase; 95% CI 0.73−0.99) were all associated with a higher risk of recurrence. When adjusted for sex and age at KF, the association with cold ischemia time was no longer significant (Table 2).

**Table 2:** Risk factors of recurrent IgAN by cox regression mode.

	Univariable		Multivariable	
Risk factors	Hazard ratio	95% CI	Hazard ratio	95% CI
**Pre-transplantation-factors**				
Sex, female (vs male)	0.99	0.37-2.72		
Active smoking	0.92	0.21-4.06	1.10	0.24-5.03
Age at time of KF^a^ (per yearly increase in age)	0.93	0.89-0.98		
Prescence of crescents in native kidney biopsy	2.00	0.71-5.61	1.44	0.49-4.19
Mesangial proliferation in native kidney biopsy	0.85	0.24-2.99	0.83	0.23-2.99
Time from diagnosis to KF (per year)	0.70	0.54-0.92	0.70	0.54-0.91
**Transplantation-factors**				
Induction therapy (basiliximab vs thymoglobulin)	1.85	0.24-14.55	3.20	0.39-26.50
Pre-emptive transplantation, yes (vs no)	1.41	0.40-4.99	1.34	0.38-4.75
Donortype:				
Genetically related (vs non-genetically related)	3.92	1.38-11.17	2.53	0.86-7.32
Living (vs deceased)	5.15	1.48-17.94	3.75	1.06-13.20
AB0 combability (vs incompability)	2.26	0.64-8.02	2.23	0.62-8.01
Donor specific antibodies positivity at time of transplantation	1.02	0.13-7.84	0.60	0.66-5.41
HLA zero-mismatch	0.53	0.07-4.12	0.76	0.10-5.90
Cold ischemia time (per hour increase in time)	0.85	0.73-0.99	0.87	0.76-1.01
**Post-Transplantation-factors**				
Acute rejection	1.32	0.43-4.01	1.60	0.51-4.99
Steroid, yes (vs no)	3.53	0.47-26.70	3.85	0.50-29.58
Steroid, post-transplant treatment years (per yearly increase)	1.02	0.93-1.12	1.02	0.93-1.13

Hazard ratios (HR) with 95% confidence intervals (CI) are presented from univariable and multivariable Cox regression analyses. The multivariable model was adjusted for sex and age at kidney failure.

^a^Kidney Failure.

Transplantation with a living donor was associated with an increased risk of recurrent IgAN compared with deceased donors (unadjusted HR 5.1; 95% CI 1.5−17.9; Table 2, Fig 2C).

A similar association was observed for genetically related donors compared with non-genetically related donors (unadjusted HR 3.9; 95% CI 1.4−11.1; Table 2, Fig 2B).

Both associations were attenuated after adjustment for sex and age at KF (Table 2).

There was no significant difference in recurrence risk between patients on steroid-free and steroid-containing immunosuppression protocols (unadjusted HR 3.5; 95% CI 0.5−26.7; adjusted HR 3.9; 95%CI 0.5−29.6, Table 2). Similarly, years on steroid treatment after transplantation did not affect recurrence risk (unadjusted HR 1.02; 95% CI 0.93−1.12, adjusted HR 1.02; 95% CI 0.93−1.13, Table 2).

No associations were found between the risk of recurrent IgAN and sex, smoking status, type of induction therapy, pre-emptive transplantation, AB0-compatibility, donor specific antibodies at the time of transplantation, HLA-zero mismatch, baseline histology, or rejection history (Table 2).

### Graft outcome and survival

In total, 28/118 recipients (24%) experienced graft failure during follow-up. Graft failure occurred in 10/17 (59%) recipients in the recurrent IgAN group and 18/101 (18%) in the non-recurrent IgAN group. Median time from recurrent IgAN to graft failure was 2.1 years (IQR 0.82−5.03). Among ten recipients with recurrent IgAN and graft failure, five failures were due to recurrence. In four cases, recurrence contributed together with infection and rejection, and in one case the cause was unknown.

A cox regression analysis examining the risk of graft failure, treating recurrent IgAN as a time-varying covariate, revealed an unadjusted HR 3.8 (95%CI 1.1−13.2) of graft failure in patients with recurrent IgAN compared with patients without. After adjusting for age at time of KF and sex the HR remained higher for patients with recurrent IgAN compared to those without (6.7, 95% CI 1.7−26.8). These findings suggest that recurrent IgAN is independently associated with graft failure.

5- and 10 years death-censored graft survival were 70% and 51% in the recurrent IgAN group and 91% and 77% in the non-recurrent IgAN group.

Death was rare in both groups: one patient with recurrent IgAN and nine without recurrence died during follow-up, five patients died with a functioning graft.

There were no differences in delayed graft function, rejections, or death comparing patients with and without recurrent IgAN (Table 1).

## Discussion

In this Danish cohort study the recurrence rate of IgAN was 14% during a median follow-up of 5.5 years, with a cumulative incidence of 16% at 10 years post-transplantation. Transplantation with a living donor, particularly a genetically related donor, was a potential risk factor for recurrence. Furthermore, recipients with recurrent IgAN were more likely to be younger at the time of diagnosis and at KF, and to have experienced shorter time interval form primary diagnosis to KF compared to those without recurrence, while post-transplant steroid use did not influence the risk of IgAN recurrence.

The recurrence rate of IgAN in this Danish cohort is consistent with similar studies based on indication biopsies to diagnose recurrent IgAN (9.9%−35.4%) [7]. In particular, the two largest published cohorts, with 2,393 and 504 patients from Europe, North and South America, and Australia and New Zealand, reported recurrence rates of 9.7% and 15% at median follow-up times of 6.1 and 8.7 years, respectively [18,22].

In the present study, the cumulative incidence of biopsy-proven recurrent IgAN was 16% at 10 years. This is consistent with a registry-based study from Australia and New Zealand including 225 IgAN recipients that reported a cumulative incidence of 10.1% at 10 years post transplantation [5], while the cohort study of 504 transplant recipients from Europe, North and South America reported incidences of 19% at 10 years and 23% at 15 years post-transplantation. Notably, when recurrence analysis in the latter study was limited to patients who underwent any post-transplant biopsy, the rate increased to 42% at 10 years [18]. Similarly, protocol biopsies in 165 recipients at the Mayo Clinic revealed recurrence rates of 12.5%, 42%, and 51% at 1, 3, and 5 years, respectively [23]. This underscores the impact of the biopsy strategy on recurrence estimates

In our cohort, as in many similar studies, allograft biopsies were performed only on clinical indication [16,24,25]. Thus, variation in reported rates and cumulative incidence across studies most likely reflects differences in biopsy practices, follow-up duration, and geographical variation in the prevalence of IgAN.

Several studies, including the present cohort, observed that recipients younger at the time of KF and progressing more rapidly to KF appear to have a higher risk of recurrence after transplantation [6,14,26,27]. In our study, each additional year of age at KF was associated with a 7% reduction in recurrence risk, consistent with data from Australia and New Zealand reporting a similar risk reduction [5]. A recent meta-analysis also indicated that patients with more rapid progression of primary disease are more susceptible to recurrence after transplantation [28]. This association may reflect a more aggressive IgAN phenotype, where rapid progression in the native kidneys predicts both earlier need for transplantation and a higher likelihood of recurrence. Potential explanations include a stronger or more persistent systemic autoimmune response, as well as age-related changes in immune function and autoantibody production [29].

The association between a living related donor and the recurrence of IgAN is controversial. Our Danish data suggest an increased risk of recurrent IgAN, when receiving a kidney from a living donor. Similar results were reported in a recent meta-analysis including 952 living related donors, a large register-based study from Australia and New Zealand including 557 living related donors and other observational studies comparable to ours [10,11,15,20]. In contrast, a multicenter study involving three centers in the USA and Portugal with 97 living and related donors found no association between living and related donors and the risk of recurrent IgAN [13]. Similarly, two studies from North America with 62 related living donors, and from Greece with 42 living related donors, respectively, did not identify any association between living and related donors and the risk of recurrent IgAN [14,24]. Potential explanations for the divergent findings include differences in indication for kidney biopsies, sample sizes, as well as racial and geographical variations among the study populations. It has been speculated that the greater risk of recurrent IgAN with living and genetically related donors may be related to a higher degree of HLA matching between donors and recipients, leading to lower immunosuppression, or to a genetically associated risk of recurrent IgAN [15]. However, we found no association between HLA matching and IgAN recurrence in our study. Larger and prospective studies are warranted to clarify this question further.

We did not identify a significant association between post-transplant steroid use and the risk of recurrent IgAN in our cohort. This is consistent with a large multicenter international study including 520 patients, 76 of whom were on steroid-free/early steroid withdrawal immunosuppressive protocol [18]. In contrast, a cohort study including 171 patients with 10 on a steroid-free protocol reported that steroid maintenance protocol was associated with a lower risk of recurrence (HR 0.37; 95% CI 0.19–0.73) [14]. This study is comparable to ours in terms of population size, but different in methodology since patients with recurrent IgAN were included from five centers, whereas patients without recurrence were only included from one center. Additionally, five protocol biopsies were included while our study did not include this. Furthermore, in both the mentioned studies and several others the indication for use of post-transplant steroid-free immunosuppression protocol is not specified [12–14,18,21]. In our study the use of steroids was determined by the prespecified immunosuppressive protocols at the two included centers, which may have reduced indication bias for the use of steroids compared to other studies in which this was solely up to the treating physician. However, the limited number of recurrence events in our cohort results in wide confidence intervals and uncertainty in the effect estimates. Therefore, the absence of a significant association should be interpreted with caution and cannot exclude a potential effect of maintenance post-transplant steroids.

We identified an increased risk of graft failure among patients with recurrent IgAN compared with those without recurrence, consistent with previous studies [18,22,27]. However, graft failure in recipients with recurrent IgAN was, often multifactorial and occurred in the context of additional processes such as rejection or infection. As graft biopsies were performed only on clinical indication, recurrent IgAN was sometimes identified during evaluation of graft dysfunction attributed to other causes. Moreover, since recurrent IgAN may be diagnosed many years post-transplant, death censored graft survival and comparisons between recurrent and non-recurrent groups are susceptible to immortal time-bias and should be interpreted with caution [30].

Strength of the present study include systematic data collection and the use of patient record review, ensuring accurate assessment of both inclusion criteria and outcomes. Furthermore, we applied strict criteria for biopsy-proven IgAN both as primary and recurrent disease. Finally, steroid use was determined by the prespecified immunosuppressive protocols, limiting indication bias, and the two transplantation centers were otherwise similar concerning ethnicity, socio-economic status, and other aspects of treatment differing only by regional residency. Some limitations need to be acknowledged. First, due to the retrospective design of the study, we cannot exclude potential selection bias and confounders. Secondly, the relatively small cohort and the limited number of recurrence events limit the conclusions, especially concerning potential weak associations. Thirdly, biopsies were performed only on clinical indications, implying that the recurrence rate of IgAN may have been underestimated, and although histopathological criteria were applied and uncertain cases re-evaluated, some degree of misclassification cannot be excluded. Finally, the Oxford MEST classification was not systematically available, as many biopsies were performed before its widespread implementation.

## Conclusions

In conclusion, recurrence of IgAN after kidney transplantation remains a clinically significant challenge, associated with younger age, rapid progression to KF, and transplantation from living or genetically related donors. Maintenance post-transplant steroid use was not associated with recurrence risk in this cohort.

Future studies with prospective designs are warranted to better define risk factors and guide post-transplant immunosuppressive strategies.
