# Supplementary material for: Incidence and risk factors for recurrent IgA nephropathy after kidney transplantation: A multicenter cohort study from Denmark
Source: PLoS One. 2026 Jul 20;21(7):e0353887. doi: 10.1371/journal.pone.0353887 (PMC13384263; doi:10.1371/journal.pone.0353887)
Supplement: Attachment — Submitted filename: Response to Reviewers.docx [file pone.0353887.s002.docx]

Dear Editor-in-chief.

PLOS One

31 March 2026

Thank you for the opportunity to resubmit our work “*Incidence and risk factors for recurrent IgA nephropathy after kidney transplantation: a multicenter cohort study from Denmark*”. We thank both reviewers for their constructive comments and suggestions, which has led to several changes, that has significantly improved the quality of the paper. In the accompanying itemized response, we have addressed all reviewer comments point-by-point and corrected in the text accordingly. We hope the adjustments are satisfactory making the paper suitable for PLOS One.

In the itemized response, reviewer comments are presented in black font, our responses are presented in red font, and specific revisions made to the manuscript are presented in *blue italic.* For each change, we have indicated the corresponding section, page, and line numbers in the revised manuscript.

Yours sincerely, on behalf of all the authors,

Mette Thrane Øhrstrøm, MD

Department of Renal Medicine

Aarhus University Hospital, Aarhus

Denmark

## Response to reviewers

**Reviewer #1:**

Thank you for the opportunity to review this manuscript regarding the incidence and risk factors for recurrent IgA nephropathy after kidney transplantation in a multicenter cohort study from Denmark. This is an interesting small retrospective multicenter analysis about recurrent IgA nephropathy after kidney transplantation in 120 recipients between 1990 and 2020. Seventeen of 120 patients had IgA nephropathy recurrence on for cause biopsies. I suggest that the analysis raises some issues in its present form and request some modifications to improve its impact and generalizability.

*Reviewer comment #1*

Can you show the changes in the Oxford classification between the first biopsy and the recurrence? It is unclear whether treatment was changed after the IgA nephropathy recurrence, and the possible impact of that in terms of time to graft failure and death.

*Response:*

A comparison of the Oxford classification between the initial biopsy and the biopsy obtained at recurrence would indeed be very interesting from, in particular from a pathophysiological and disease-understanding perspective.

However, the Oxford classification was not systematically reported in pathology records throughout the study period (1990–2020), as many biopsies were performed before widespread implementation of the Oxford classification. Therefore, data were not consistently reported in the historical pathology reports. As we do not have access to all biopsy specimens, we unfortunately are not able to perform a reevaluation. We have clarified this in the methods and added it as a limitation in the discussion section of the revised manuscript.

The immunosuppressive protocol was not change after IgA nephropathy recurrence as per local guidelines and clinical practice, although conservative management, including renin–angiotensin system inhibitors may have been introduced.

*Manuscript changes #1*

Methods section (page 6, lines 110-112)

“The date of the graft biopsy defined the time of recurrence. All kidney biopsies (native or graft) were clinically indicated; no protocol biopsies were performed. *The Oxford MEST classification was not systematically reported in the pathology records throughout the study period and was therefore not available for consistent analysis in this cohort.”*

Discussion section (page 18, lines 343-344)

“Secondly, the relatively small cohort and the limited number of recurrence events limit the conclusions, especially concerning potential weak associations. Thirdly, biopsies were performed only on clinical indications, implying that the recurrence rate of IgAN may have been underestimated, and although histopathological criteria were applied and uncertain cases re-evaluated, some degree of misclassification cannot be excluded. *Finally, the Oxford MEST classification was not systematically available, as many biopsies were performed before its widespread implementation.”*

*Reviewer comment #2*

Four out of ten who developed graft failure presented IgA nephropathy recurrence in combination with infection and rejection. How many of them have graft loss due to IgA or rejection/infection? It would be more appropriate to perform an analysis excluding these patients if the primary cause was not IgA recurrence. Additionally, in Uffing et al. (ref#18, 10.2215/CJN.00910121), IgA nephropathy recurrence rates were higher in patients with de novo DSA and pre-emptive. Can the authors comment on that and report their data about DSAs in their population?

*Response:*

Thank you for the suggestion, which is indeed relevant; however, restricting the analysis to graft failures clearly attributable to recurrent IgAN would reduce the number of events to five, resulting in unstable estimates with wide confidence intervals and limited interpretability. Instead, we have revised the wording in the manuscript and clarified in the discussion that graft loss in recipients with recurrent IgAN was frequently multifactorial and that the relative contribution of recurrent IgAN versus other processes such as rejection or infection may be difficult to determine.

In addition, graft biopsies in our cohort were performed only on clinical indication, typically due to suspected rejection or graft dysfunction. Recurrent IgAN could therefore be detected during evaluation of graft dysfunction related to other causes. We have clarified this point in the discussion section of the revised manuscript.

We also thank the reviewer for highlighting the important study by Uffing et al. To address this point, we have now included pre-emptive transplantation in our analyses. Donor-specific antibodies at the time of transplantation were already part of the dataset and analysis. In our cohort, neither pre-emptive transplantation nor donor-specific antibodies at the time of transplantation were associated with recurrent IgAN. Data on the development of de novo DSA during follow-up were not systematically available throughout the study period and could therefore not be analyzed reliably in this cohort. Differences between our findings and those reported by Uffing et al. may relate to differences in cohort size and statistical power, as well as the lack of systematic data on de novo DSA in our cohort.

*Manuscript changes #2*

Result section (page 13, line 235)

“These findings suggest that recurrent IgAN is *independently associated* with graft failure. “

Discussion section (page 17, lines 322-326)

“We identified an increased risk of graft failure among patients with recurrent IgAN compared with those without recurrence, consistent with previous studies [18, 22, 27]. *However,* *graft failure in recipients with recurrent IgAN was, however, often multifactorial and occurred in the context of additional processes such as rejection or infection. As graft biopsies were performed only on clinical indication, recurrent IgAN was sometimes identified during evaluation of graft dysfunction attributed to other causes.* Moreover, since recurrent IgAN may be diagnosed many years post-transplant, death censored graft survival and comparisons between recurrent and non-recurrent groups are susceptible to immortal time-bias and should be interpreted with caution [30]”

Pre-emptive transplantation has now been added to Table 1 and included in the risk factor analysis in Table 2. Donor-specific antibodies at the time of transplantation were already included in the analysis. These variables have also been added to the corresponding description in the Results section (page 10, line 181 and page 12, line 216).

*Reviewer comment #3*

Data about IgA recurrence treatment should at least be presented in supplementary material and commented on.

*Response*

Treatment of recurrent IgAN was not systematically recorded, as it was not a predefined aim of the study. According to local guidelines and clinical practice, immunosuppressive therapy was not modified, although conservative management, including renin–angiotensin system inhibitors, may have been introduced.

*Reviewer comment #4*

Complement activation is acquiring greater importance in IgA nephropathy, and some new drugs targeting complement pathways (e.g., iptacopan, pegcetacoplan) showed promising results. Recent findings suggest that complement deposition could be abrogated in post-treatment biopsies (see 10.1016/j.ajt.2025.08.015). Could complement deposition (at least C3, ideally C5b-9) be evaluated in the patient and commented on the eventual differences or changes between the steroid-free group and the steroid group?

*Response*

We fully agree that complement activation is increasingly recognized as an important mechanism in IgA nephropathy. Thus, the suggestion to look at complement staining is indeed interesting; however, beyond the primary scope of the paper. Unfortunately, complement deposition was not systematically recorded in the pathology reports for our cohort and we did not have access to a systematic re-evaluation of all biopsy specimens.

*Reviewer comment #5*

I do not fully understand if the differences among patients with recurrent IgAN (younger at time of IgAN diagnosis, younger at KF, faster progression from primary diagnosis to KF were significant or only trends. Please include the p-value in the text and tables.

*Respons*

We are sorry that this was not clear. We have now added p-values to Table 1, page 9-10, and to the corresponding description in the results section, page 8. In addition, the p-value has been added to Figure 2. Table 2 presents the results of the Cox regression analyses with hazard ratios and 95% confidence intervals; statistical significance can therefore be inferred from whether the confidence intervals include 1.

*Reviewer comment #6*

Other induction therapy refers to no induction, steroids only, or alemtuzumab? Please specify.

*Response*

We have clarified this point in the revised manuscript. The category “other” is now specified in the note below Table 1, page 10.

*Reviewer comment #7*

Minor point: Correct typos in Table 1 (e.g., Immunodeposits on electron microscopy 74(120) 62%)

*Response*

Thank you for noticing this. We have corrected the typographical errors in Table 1, page 9

**Reviewer #2:**

**Major comments**

*Reviewer comment #1*

Disease definition and potential misclassification (IgA nephropathy).

The definition of IgA nephropathy requires clarification. In addition to cases with predominant mesangial IgA deposition on immunofluorescence, the manuscript includes patients described as having “mesangioproliferative glomerulonephritis where IgAN was most likely.” As mesangioproliferative GN represents a histopathological pattern rather than a specific diagnosis, this definition may lack specificity. In particular, other conditions such as fibrillary glomerulonephritis can present with a mesangioproliferative pattern and may occasionally show IgA or mixed immunoglobulin deposition. The use of the term “most likely IgAN” is subjective and may reduce reproducibility, especially when the same criteria are applied to define recurrent disease in allograft biopsies, where mesangioproliferative changes are often non-specific.

The authors are encouraged to clarify the exact histopathological criteria used to define both primary and recurrent IgAN, including whether electron microscopy was systematically performed and whether fibrillary or other non-IgA immune complex glomerulopathies were explicitly excluded. Clarifying these points would strengthen confidence in the reported recurrence rates and subsequent risk factor analyses.

*Response*

We agree that the original definition of IgA nephropathy (IgAN), including the term “most likely IgAN,” lacked specificity.

In the revised manuscript, we have now clarified and refined the histopathological definition of IgAN. To include kidney biopsies with either predominant mesangial IgA deposition on immunofluorescence and/or a mesangioproliferative glomerulonephritis with immune complex deposits identified on electron microscopy.

Cases with inconclusive findings were re-evaluated. Following this process, two patients were excluded from the cohort, as the diagnosis could not be supported by these criteria. All analyses were therefore repeated in the updated cohort (n=118). Although numerical estimates changed slightly, the overall conclusions remained unchanged.

These clarifications have been incorporated into the Methods section (page 6, lines 104-106), and the potential for residual misclassification is addressed in the Limitations (page 18, lines 338-340).

*Reviewer comment #2*

Interpretation of steroid analysis and statistical considerations.

The analysis of maintenance steroid use is limited by the small number of recurrence events, resulting in wide confidence intervals and limited statistical precision. Although the lack of statistical significance is appropriately reported, the discussion currently presents the steroid findings in a relatively definitive manner. Given the imprecision of the estimates, the absence of a statistically significant association should be interpreted with caution and should not be equated with evidence of no effect.

This issue would benefit from being addressed explicitly in the discussion, rather than only as a general limitation, by emphasizing limited power and uncertainty in the effect estimates. A more cautious wording would better reflect the statistical constraints of the dataset.

*Response*

We have revised the discussion of the steroid analysis to emphasize the uncertainty in the effect estimates and the need for cautious interpretation. We have also clarified this point in the general limitations section.

*Manuscript changes #2*

Discussion section (page 17, lines 315-319)

“In our study the use of steroids was determined by the prespecified immunosuppressive protocols at the two included centers, which may have reduced indication bias for the use of steroids compared to other studies in which this was solely up to the treating physician. *However, the limited number of recurrence events in our cohort results in wide confidence intervals and uncertainty in the effect estimates. Therefore, the absence of a significant association should be interpreted with caution and cannot exclude a potential effect of maintenance post-transplant steroids.”*

Discussion section (page 18, line 338-340)

“*Secondly, the relatively small cohort and the limited number of recurrence events limit the conclusions, especially concerning potential weak associations.*”

**Minor comments**

*Reviewer comment #1*

Figure 1 contains a labeling error in which the recurrent and non-recurrent IgAN groups appear to be inverted, which conflicts with the text and Table 1 and should be corrected. Figure 2 is of suboptimal visual quality and appears blurred; higher-resolution images would improve readability. In addition, the tables contain a small number of typographical and formatting issues that should be corrected for clarity and consistency.

*Response #1*

Thank you for pointing to these. The labelling in Figure 1 has been corrected, Figure 2 has been replaced with a higher-resolution version, and minor typographical and formatting issues in the tables have been corrected.
