# Supplementary material for: Incidence and risk factors for recurrent IgA nephropathy after kidney transplantation: A multicenter cohort study from Denmark
Source: PLoS One. 2026 Jul 20;21(7):e0353887. doi: 10.1371/journal.pone.0353887 (PMC13384263; doi:10.1371/journal.pone.0353887)
Supplement: Peer review history

# Peer review: Decision Letter 0

**DOI:** 10.1371/journal.pone.0353887.r001

**Authors:** Jeevithan Elango

11 Feb 2026

PONE-D-25-66090

Incidence and risk factors for recurrent IgA nephropathy after kidney transplantation: a multicenter cohort study from Denmark

PLOS One

Dear Dr. Øhrstrøm,

Thank you for submitting your manuscript to PLOS ONE. After careful consideration, we feel that it has merit but does not fully meet PLOS ONE’s publication criteria as it currently stands. Therefore, we invite you to submit a revised version of the manuscript that addresses the points raised during the review process.

==============================

**ACADEMIC EDITOR:**

The manuscript is novel and provides new insights to the field. However, there are major concerns as raised by the reviewers. Hence, i request to revise the MS based on the reviewers comments and resubmit for further consideration.

==============================

Please submit your revised manuscript by Mar 28 2026 11:59PM. If you will need more time than this to complete your revisions, please reply to this message or contact the journal office at plosone@plos.org. When you're ready to submit your revision, log on to https://www.editorialmanager.com/pone/ and select the 'Submissions Needing Revision' folder to locate your manuscript file.

Please include the following items when submitting your revised manuscript:

- A letter that responds to each point raised by the academic editor and reviewer(s). You should upload this letter as a separate file labeled 'Response to Reviewers'.
- A marked-up copy of your manuscript that highlights changes made to the original version. You should upload this as a separate file labeled 'Revised Manuscript with Track Changes'.
- An unmarked version of your revised paper without tracked changes. You should upload this as a separate file labeled 'Manuscript'.

If you would like to make changes to your financial disclosure, please include your updated statement in your cover letter. Guidelines for resubmitting your figure files are available below the reviewer comments at the end of this letter.

If applicable, we recommend that you deposit your laboratory protocols in protocols.io to enhance the reproducibility of your results. Protocols.io assigns your protocol its own identifier (DOI) so that it can be cited independently in the future. For instructions see: https://journals.plos.org/plosone/s/submission-guidelines#loc-laboratory-protocols. Additionally, PLOS ONE offers an option for publishing peer-reviewed Lab Protocol articles, which describe protocols hosted on protocols.io. Read more information on sharing protocols at https://plos.org/protocols?utm_medium=editorial-email&utm_source=authorletters&utm_campaign=protocols.

We look forward to receiving your revised manuscript.

Kind regards,

Jeevithan Elango, PhD

Academic Editor

PLOS One

Journal requirements:

When submitting your revision, we need you to address these additional requirements.

1. Please ensure that your manuscript meets PLOS ONE's style requirements, including those for file naming. The PLOS ONE style templates can be found at

https://journals.plos.org/plosone/s/file?id=wjVg/PLOSOne_formatting_sample_main_body.pdf and

https://journals.plos.org/plosone/s/file?id=ba62/PLOSOne_formatting_sample_title_authors_affiliations.pdf

2. We note that you have indicated that there are restrictions to data sharing for this study. For studies involving human research participant data or other sensitive data, we encourage authors to share de-identified or anonymized data. However, when data cannot be publicly shared for ethical reasons, we allow authors to make their data sets available upon request. For information on unacceptable data access restrictions, please see http://journals.plos.org/plosone/s/data-availability#loc-unacceptable-data-access-restrictions.

Before we proceed with your manuscript, please address the following prompts:

a) If there are ethical or legal restrictions on sharing a de-identified data set, please explain them in detail (e.g., data contain potentially identifying or sensitive patient information, data are owned by a third-party organization, etc.) and who has imposed them (e.g., a Research Ethics Committee or Institutional Review Board, etc.). Please also provide contact information for a data access committee, ethics committee, or other institutional body to which data requests may be sent.

b) If there are no restrictions, please upload the minimal anonymized data set necessary to replicate your study findings to a stable, public repository and provide us with the relevant URLs, DOIs, or accession numbers. Please see http://www.bmj.com/content/340/bmj.c181.long for guidelines on how to de-identify and prepare clinical data for publication. For a list of recommended repositories, please see https://journals.plos.org/plosone/s/recommended-repositories. You also have the option of uploading the data as Supporting Information files, but we would recommend depositing data directly to a data repository if possible.

Please update your Data Availability statement in the submission form accordingly.

3. We notice that your supplementary figures are uploaded with the file type 'Figure'. Please amend the file type to 'Supporting Information'. Please ensure that each Supporting Information file has a legend listed in the manuscript after the references list.

If the reviewer comments include a recommendation to cite specific previously published works, please review and evaluate these publications to determine whether they are relevant and should be cited. There is no requirement to cite these works unless the editor has indicated otherwise.

Additional Editor Comments:

Dear Author,

Please revise your MS based on the reviewers' comments and resubmit.

[Note: HTML markup is below. Please do not edit.]

Reviewers' comments:

Reviewer's Responses to Questions

**Comments to the Author**

1. Is the manuscript technically sound, and do the data support the conclusions?

The manuscript must describe a technically sound piece of scientific research with data that supports the conclusions. Experiments must have been conducted rigorously, with appropriate controls, replication, and sample sizes. The conclusions must be drawn appropriately based on the data presented.

Reviewer #1: Partly

Reviewer #2: Yes

**********

2. Has the statistical analysis been performed appropriately and rigorously?

Reviewer #1: Yes

Reviewer #2: Yes

**********

3. Have the authors made all data underlying the findings in their manuscript fully available?

The PLOS Data policy requires authors to make all data underlying the findings described in their manuscript fully available without restriction, with rare exception (please refer to the Data Availability Statement in the manuscript PDF file). The data should be provided as part of the manuscript or its supporting information, or deposited to a public repository. For example, in addition to summary statistics, the data points behind means, medians and variance measures should be available. If there are restrictions on publicly sharing data—e.g. participant privacy or use of data from a third party—those must be specified.

Reviewer #1: Yes

Reviewer #2: Yes

**********

4. Is the manuscript presented in an intelligible fashion and written in standard English?

PLOS ONE does not copyedit accepted manuscripts, so the language in submitted articles must be clear, correct, and unambiguous. Any typographical or grammatical errors should be corrected at revision, so please note any specific errors here.

Reviewer #1: Yes

Reviewer #2: Yes

**********

5. Review Comments to the Author

Please use the space provided to explain your answers to the questions above. You may also include additional comments for the author, including concerns about dual publication, research ethics, or publication ethics. (Please upload your review as an attachment if it exceeds 20,000 characters)

Reviewer #1: Thank you for the opportunity to review this manuscript regarding the incidence and risk factors for recurrent IgA nephropathy after kidney transplantation in a multicenter cohort study from Denmark. This is an interesting small retrospective multicenter analysis about recurrent IgA nephropathy after kidney transplantation in 120 recipients between 1990 and 2020. Seventeen of 120 patients had IgA nephropathy recurrence on for cause biopsies. I suggest that the analysis raises some issues in its present form and request some modifications to improve its impact and generalizability.

1. Can you show the changes in the Oxford classification between the first biopsy and the recurrence? It is unclear whether treatment was changed after the IgA nephropathy recurrence, and the possible impact of that in terms of time to graft failure and death.

2. Four out of ten who developed graft failure presented IgA nephropathy recurrence in combination with infection and rejection. How many of them have graft loss due to IgA or rejection/infection? It would be more appropriate to perform an analysis excluding these patients if the primary cause was not IgA recurrence. Additionally, in Uffing et al. (ref#18, 10.2215/CJN.00910121), IgA nephropathy recurrence rates were higher in patients with de novo DSA and pre-emptive. Can the authors comment on that and report their data about DSAs in their population?

3. Data about IgA recurrence treatment should at least be presented in supplementary material and commented on.

4. Complement activation is acquiring greater importance in IgA nephropathy, and some new drugs targeting complement pathways (e.g., iptacopan, pegcetacoplan) showed promising results. Recent findings suggest that complement deposition could be abrogated in post-treatment biopsies (see 10.1016/j.ajt.2025.08.015). Could complement deposition (at least C3, ideally C5b-9) be evaluated in the patient and commented on the eventual differences or changes between the steroid-free group and the steroid group?

5. I do not fully understand if the differences among patients with recurrent IgAN (younger at time of IgAN diagnosis, younger at KF, faster progression from primary diagnosis to KF were significant or only trends. Please include the p-value in the text and tables.

6. Other induction therapy refers to no induction, steroids only, or alemtuzumab? Please specify.

7. Minor point: Correct typos in table 1 (e.g., Immunodeposits on electron microscopy 74(120) 62%)

Reviewer #2: Major comments

1. Disease definition and potential misclassification (IgA nephropathy)

The definition of IgA nephropathy requires clarification. In addition to cases with predominant mesangial IgA deposition on immunofluorescence, the manuscript includes patients described as having “mesangioproliferative glomerulonephritis where IgAN was most likely.” As mesangioproliferative GN represents a histopathological pattern rather than a specific diagnosis, this definition may lack specificity. In particular, other conditions such as fibrillary glomerulonephritis can present with a mesangioproliferative pattern and may occasionally show IgA or mixed immunoglobulin deposition. The use of the term “most likely IgAN” is subjective and may reduce reproducibility, especially when the same criteria are applied to define recurrent disease in allograft biopsies, where mesangioproliferative changes are often non-specific.

The authors are encouraged to clarify the exact histopathological criteria used to define both primary and recurrent IgAN, including whether electron microscopy was systematically performed and whether fibrillary or other non-IgA immune complex glomerulopathies were explicitly excluded. Clarifying these points would strengthen confidence in the reported recurrence rates and subsequent risk factor analyses.

2. Interpretation of steroid analysis and statistical considerations

The analysis of maintenance steroid use is limited by the small number of recurrence events, resulting in wide confidence intervals and limited statistical precision. Although the lack of statistical significance is appropriately reported, the discussion currently presents the steroid findings in a relatively definitive manner. Given the imprecision of the estimates, the absence of a statistically significant association should be interpreted with caution and should not be equated with evidence of no effect.

This issue would benefit from being addressed explicitly in the discussion, rather than only as a general limitation, by emphasizing limited power and uncertainty in the effect estimates. A more cautious wording would better reflect the statistical constraints of the dataset.

Minor comments

Figure 1 contains a labeling error in which the recurrent and non-recurrent IgAN groups appear to be inverted, which conflicts with the text and Table 1 and should be corrected. Figure 2 is of suboptimal visual quality and appears blurred; higher-resolution images would improve readability. In addition, the tables contain a small number of typographical and formatting issues that should be corrected for clarity and consistency.

**********

6. PLOS authors have the option to publish the peer review history of their article (what does this mean?). If published, this will include your full peer review and any attached files.

If you choose “no”, your identity will remain anonymous but your review may still be made public.

**Do you want your identity to be public for this peer review?**  For information about this choice, including consent withdrawal, please see our Privacy Policy.

Reviewer #1: No

Reviewer #2: No

**********

[NOTE: If reviewer comments were submitted as an attachment file, they will be attached to this email and accessible via the submission site. Please log into your account, locate the manuscript record, and check for the action link "View Attachments". If this link does not appear, there are no attachment files.]

To ensure your figures meet our technical requirements, please review our figure guidelines: https://journals.plos.org/plosone/s/figures

You may also use PLOS’s free figure tool, NAAS, to help you prepare publication quality figures: https://journals.plos.org/plosone/s/figures#loc-tools-for-figure-preparation.

NAAS will assess whether your figures meet our technical requirements by comparing each figure against our figure specifications.

# Author comment: Author response to Decision Letter 1

**DOI:** 10.1371/journal.pone.0353887.r002

12 Apr 2026

We thank the editor and reviewers for their constructive and insightful comments. We have carefully revised the manuscript accordingly and believe that these changes have improved the clarity and robustness of the study.

In particular, we have clarified the definition of IgA nephropathy, re-evaluated selected cases leading to an updated cohort (N=118). Figures have been updated to meet journal requirements, and the Data Availability statement has been revised to clarify data access in accordance with Danish legislation and the study approval by the Region of Southern Denmark.

All reviewer comments have been addressed point-by-point in the accompanying response-to-reviewers document, and all requested revisions have been incorporated into the manuscript.

Attachment Submitted filename: Response to Reviewers.docx

# Peer review: Decision Letter 1

**DOI:** 10.1371/journal.pone.0353887.r003

**Authors:** Nasar Alwahaibi

30 Jun 2026

Incidence and risk factors for recurrent IgA nephropathy after kidney transplantation: a multicenter cohort study from Denmark

PONE-D-25-66090R1

Dear Dr. Øhrstrøm,

We’re pleased to inform you that your manuscript has been judged scientifically suitable for publication and will be formally accepted for publication once it meets all outstanding technical requirements.

Within one week, you’ll receive an e-mail detailing the required amendments. When these have been addressed, you’ll receive a formal acceptance letter and your manuscript will be scheduled for publication.

An invoice will be generated when your article is formally accepted. Please note, if your institution has a publishing partnership with PLOS and your article meets the relevant criteria, all or part of your publication costs will be covered. Please make sure your user information is up-to-date by logging into Editorial Manager at Editorial Manager® and clicking the ‘Update My Information' link at the top of the page. For questions related to billing, please contact billing support.

If your institution or institutions have a press office, please notify them about your upcoming paper to help maximize its impact. If they’ll be preparing press materials, please inform our press team as soon as possible -- no later than 48 hours after receiving the formal acceptance. Your manuscript will remain under strict press embargo until 2 pm Eastern Time on the date of publication. For more information, please contact onepress@plos.org.

Kind regards,

Nasar Alwahaibi, PhD

Academic Editor

PLOS One

Reviewers' comments:

Reviewer's Responses to Questions

**Comments to the Author**

1. If the authors have adequately addressed your comments raised in a previous round of review and you feel that this manuscript is now acceptable for publication, you may indicate that here to bypass the “Comments to the Author” section, enter your conflict of interest statement in the “Confidential to Editor” section, and submit your "Accept" recommendation.

Reviewer #1: All comments have been addressed

**********

2. Is the manuscript technically sound, and do the data support the conclusions?

The manuscript must describe a technically sound piece of scientific research with data that supports the conclusions. Experiments must have been conducted rigorously, with appropriate controls, replication, and sample sizes. The conclusions must be drawn appropriately based on the data presented.

Reviewer #1: Partly

**********

3. Has the statistical analysis been performed appropriately and rigorously?

Reviewer #1: Yes

**********

4. Have the authors made all data underlying the findings in their manuscript fully available?

The PLOS Data policy requires authors to make all data underlying the findings described in their manuscript fully available without restriction, with rare exception (please refer to the Data Availability Statement in the manuscript PDF file). The data should be provided as part of the manuscript or its supporting information, or deposited to a public repository. For example, in addition to summary statistics, the data points behind means, medians and variance measures should be available. If there are restrictions on publicly sharing data—e.g. participant privacy or use of data from a third party—those must be specified.

Reviewer #1: Yes

**********

5. Is the manuscript presented in an intelligible fashion and written in standard English?

PLOS ONE does not copyedit accepted manuscripts, so the language in submitted articles must be clear, correct, and unambiguous. Any typographical or grammatical errors should be corrected at revision, so please note any specific errors here.

Reviewer #1: Yes

**********

6. Review Comments to the Author

Please use the space provided to explain your answers to the questions above. You may also include additional comments for the author, including concerns about dual publication, research ethics, or publication ethics. (Please upload your review as an attachment if it exceeds 20,000 characters)

Reviewer #1: No further revisions are required. However, the manuscript's overall impact is low due to intrinsic limitations (i.e., no evaluation of the Oxford classification and complement deposition on kidney biopsies).

**********

7. PLOS authors have the option to publish the peer review history of their article (what does this mean?). If published, this will include your full peer review and any attached files.

If you choose “no”, your identity will remain anonymous but your review may still be made public.

**Do you want your identity to be public for this peer review?**  For information about this choice, including consent withdrawal, please see our Privacy Policy.

Reviewer #1: No

**********

# Editor report: Acceptance letter

**DOI:** 10.1371/journal.pone.0353887.r004

**Authors:** Nasar Alwahaibi

PONE-D-25-66090R1

PLOS One

Dear Dr. Øhrstrøm,

I'm pleased to inform you that your manuscript has been deemed suitable for publication in PLOS One. Congratulations! Your manuscript is now being handed over to our production team.

At this stage, our production department will prepare your paper for publication. This includes ensuring the following:

* All references, tables, and figures are properly cited

* All relevant supporting information is included in the manuscript submission,

* There are no issues that prevent the paper from being properly typeset

You will receive further instructions from the production team, including instructions on how to review your proof when it is ready. Please keep in mind that we are working through a large volume of accepted articles, so please give us a few days to review your paper and let you know the next and final steps.

Lastly, if your institution or institutions have a press office, please let them know about your upcoming paper now to help maximize its impact. If they'll be preparing press materials, please inform our press team within the next 48 hours. Your manuscript will remain under strict press embargo until 2 pm Eastern Time on the date of publication. For more information, please contact onepress@plos.org.

You will receive an invoice from PLOS for your publication fee after your manuscript has reached the completed accept phase. If you receive an email requesting payment before acceptance or for any other service, this may be a phishing scheme. Learn how to identify phishing emails and protect your accounts at https://explore.plos.org/phishing.

If we can help with anything else, please email us at customercare@plos.org.

Thank you for submitting your work to PLOS ONE and supporting open access.

Kind regards,

PLOS ONE Editorial Office Staff

on behalf of

Dr. Nasar Alwahaibi

Academic Editor

PLOS One
